# *BRAF V600E-*mutated lung adenocarcinoma with thyroid metastasis as the initial manifestation: a case report

**DOI:** 10.3389/fonc.2025.1468233

**Published:** 2025-02-06

**Authors:** Yufei Liu, Hanhan Lei, Lingling Cai, Yuyan Tan, Xinyu Song

**Affiliations:** ^1^ Institute of Pathology, China Three Gorges University, Yichang, China; ^2^ Department of Pathology, Yichang Central People’s Hospital, Yichang, Hubei, China; ^3^ Department of Thyroid and breast Surgery, Yichang Central People’s Hospital, Yichang, China; ^4^ Department of Respiratory and Critical Care Medicine, Yichang Central People’s Hospital, Yichang, China

**Keywords:** non-small cell lung cancer (NSCLC), *BRAF V600E* mutation, immunohistochemistry, thyroid, metastasis

## Abstract

Thyroid metastasis of lung adenocarcinoma is exceedingly uncommon. Here we present a case of a 72-year-old Chinese male with hoarseness, dysphagia, pain, and palpable thyroid nodules. Ultrasonography-guided thyroid fine-needle aspiration cytology (FNAC) suggested a high-grade follicular-derived thyroid carcinoma (HGFCTC). Molecular analysis identified a *BRAF V600E* mutation. Comprehensive histopathological and immunohistochemical examinations, however, revealed that the thyroid cancer originated from the left lung. The patient received a 6-month post-operative dual-target therapy with dalafenib and trametinib. As of the last follow-up, the patient was still alive, demonstrating the effectiveness of targeted therapy.

## Introduction

Despite the rich vascularization, the thyroid is infrequently invaded by metastatic tumors, with an incidence as low as 0.1% (1, 2). Secondary involvement of the thyroid (SIT) can result from proximal invasion or distant metastasis. Autopsies suggest that SIT often originates from cancers with primary sites in the lung, breast, kidney, and colorectum. Thyroid metastasis of renal cancer is the most frequent, followed by colorectal and lung cancers (3–5). Accurate diagnosis of SIT is vital for cancer management. FNAC is a reliable, minimally invasive, and cost-efficient diagnostic tool for thyroid nodules (6–10). However, FNAC alone would misinterpret metastatic cancers as primary thyroid cancer, especially when the diagnosis of the primary cancers fails. However, it is challenging to identify SIT at the early stage due to the lack of specific symptoms, similarity in imaging features, and the overlapping cellular morphological characteristics between some metastatic malignant tumors and primary thyroid cancer. This case report illustrates a misdiagnosed primary thyroid cancer which was later identified as an atypical metastasis of *BRAF V600E-*mutant lung adenocarcinoma.

## Case report

**Figure 1:**
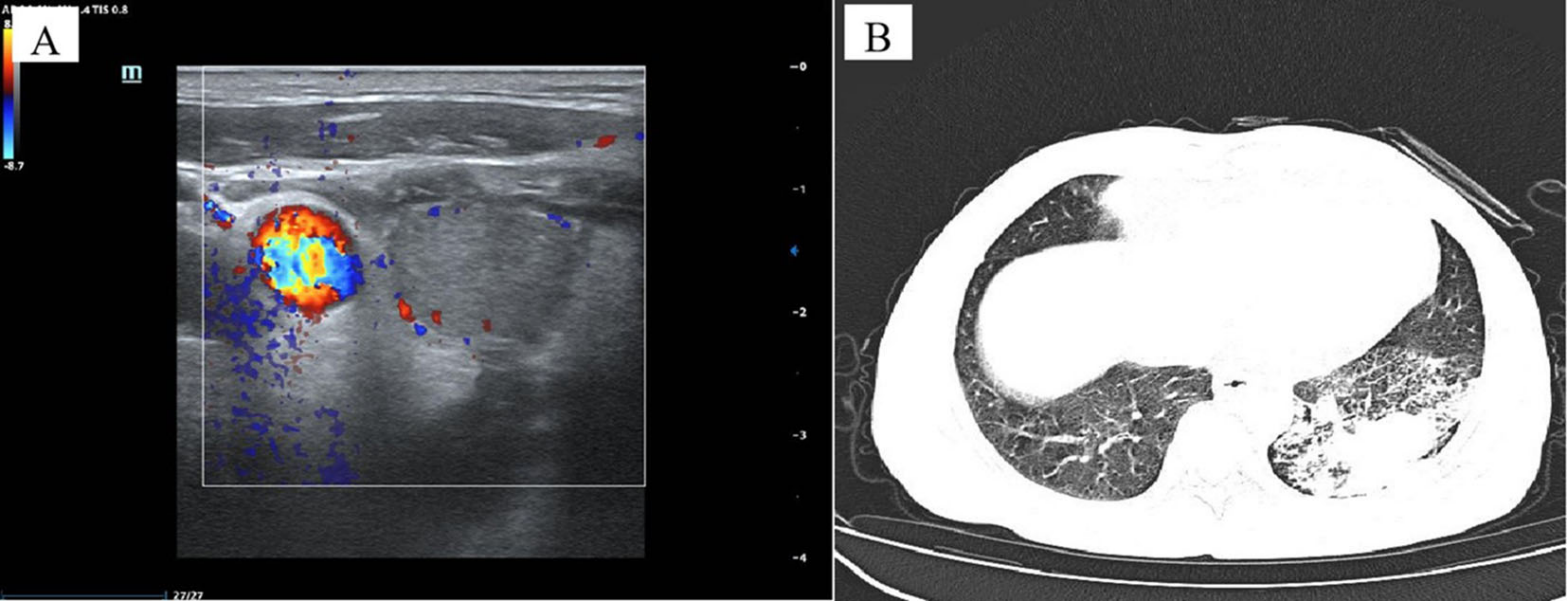
Imaging data of the case. Ultrasonography of the right thyroid lobe showed an irregular, hypoechoic nodule. Doppler ultrasound showed intra-nodular low vascularization **(A)**. Chest Computed Tomography (CT) scan showed patchy soft tissue density shadows in the left lower lung, resembling pneumonia **(B)**.

**Figure 2:**
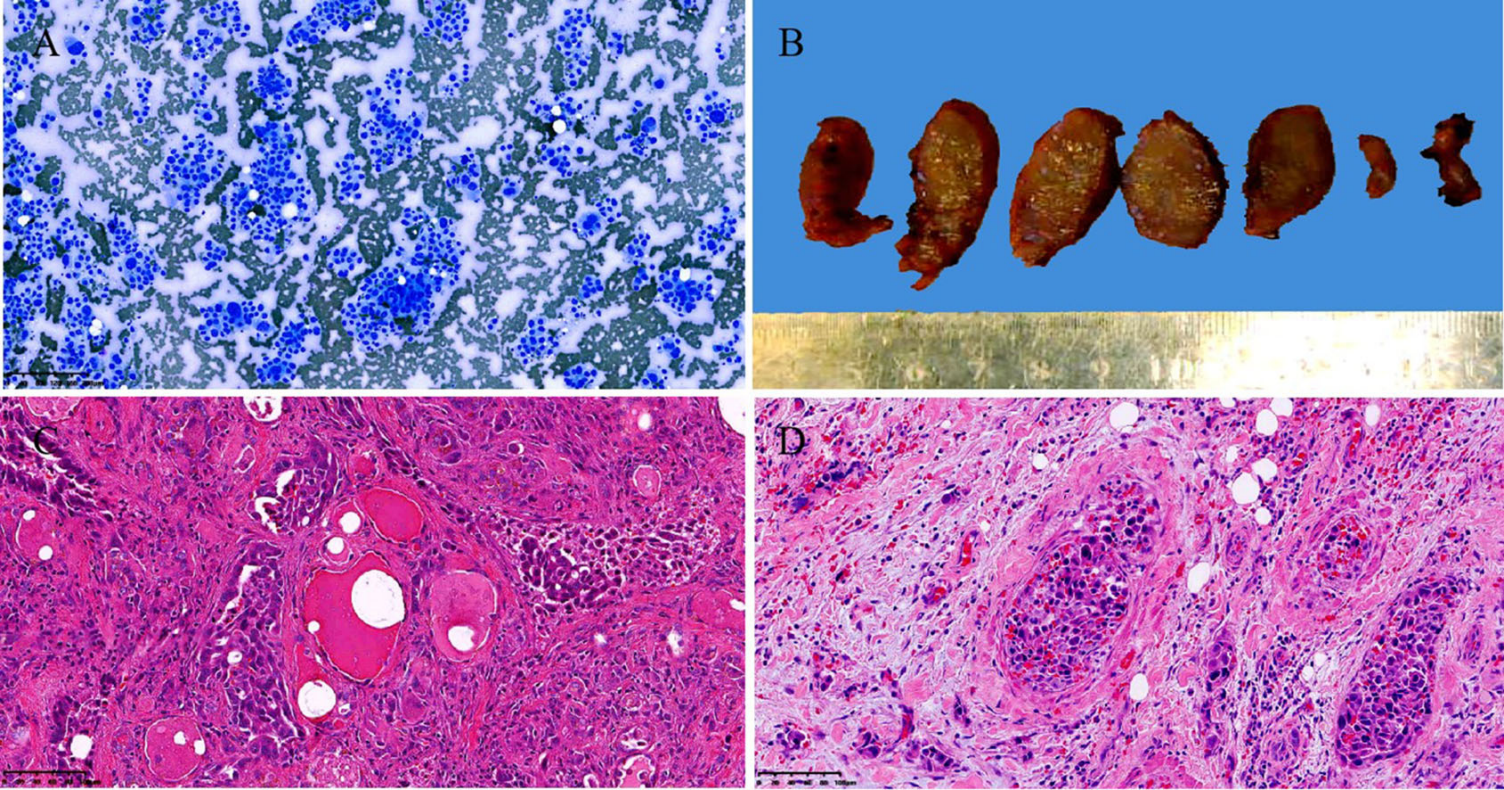
Cytological, gross pathological and histological images of the case. Thyroid fine needle aspiration cytology examination showed abundant fragments of loosely cohesive epithelial cells with a moderate amount of basophilic cytoplasm and moderate nuclear pleomorphism in a bloody background. smear, Diff-Quik staining, Original magnification: 100× **(A)**. Gross examination of the resected right thyroid gland specimen exhibited an ill-defined, solid, gray-white lesion measured 2.3cm × 1.4cm × 1.3cm **(B)**. Histopathological examination revealed that significantly atypical epithelial tumor cells infiltrating the surrounding normal thyroid follicular structure exhibited a mixed structural pattern, including acini, fused glands, cribriform, solid, and focal micropapillary structures. hematoxylin and eosin staining, Original magnification: 100× **(C)**. The extensive angiolymphatic invasion was noted in the thyroid tissue. hematoxylin and eosin staining, Original magnification: 200× **(D)**.

**Figure 3:**
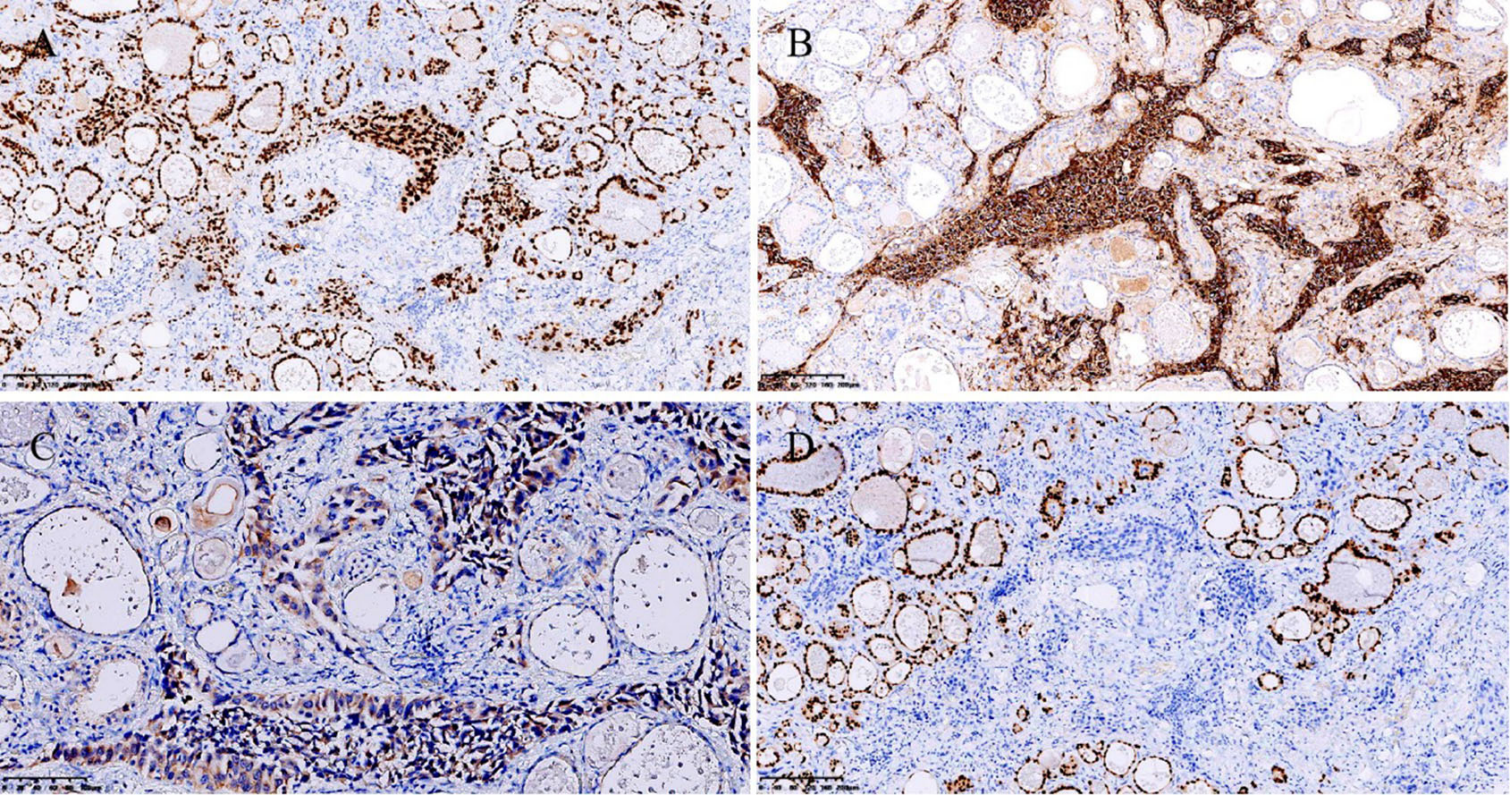
Immunohistochemistry. Strongly and diffusely positive TTF-1 staining. Original magnification: 100×. **(A)** Strongly and diffusely positive Napsin A staining. Original magnification: 100×. **(B)** Moderately positive cytoplasmic staining of BRAF V600E (clone VE1). Original magnification: 200×. **(C)** PAX8 was negative. Original magnification: 100× **(D)**.

**Figure 4:**
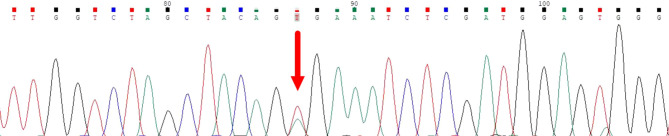
Sanger sequencing confirmed the *BRAF V600E* mutation in both lung and thyroid lesions.

A 72-year-old Chinese man with a smoking history had hoarseness, difficulty swallowing, pain, and palpable thyroid nodules without other remarkable health problems. Thyroid function was generally normal except for slightly lower free triiodothyronine. Laboratory tests showed elevated serum carcinoembryonic antigen (CEA, 73.9 ng/ml). A thyroid ultrasound showed a hypoechoic solid nodule (23.0 mm x 14.0mm in size) with irregular boundaries in the right thyroid lobe (**Figure 1A**). Lymph node enlargement was seen in the cervical and central regions. A Chest Computed Tomography (CT) scan showed pneumonia-like patchy soft tissue density shadows in the left lower lung (**Figure 1B**). The Diff-Quik (DQ) staining FNAC smears of the right thyroid nodule showed abundant fragments of loosely cohesive epithelial cells with a moderate amount of basophilic cytoplasm and moderate nuclear pleomorphism in a bloody background. These dysplastic epithelial cells were arranged in sheets and clusters or individually dispersed, exhibiting a relatively high nucleus-cytoplasm ratio. The cell clusters were relatively loose and the cell sheets were in acini, tubule, tubulopapillary, or tubulocribriform forms. No intracytoplasmic mucin and psammoma body was observed. Due to the patient presenting with thyroid nodules as the initial clinical manifestation, denying any history of other non-thyroid malignancies, and no relevant examinations indicating the presence of other non thyroid malignant tumors, FNAC diagnosed this case as high-grade follicular-derived thyroid carcinoma (HGFCTC) (**Figure 2A**). The patient then received a total thyroidectomy with lymph node dissection. The color of the resected specimen was grayish-red to grayish-white on the cut surface. Notably, an ill-defined, solid, gray-white lesion of 2.3cm × 1.4 cm × 1.3 cm was observed at the lower pole of the right thyroid lobe (**Figure 2B**). The dysplastic epithelial cells were morphologically diverse, exhibiting a mixture of the acinar, fused gland, cribriform, solid, and focal micropapillary structures. The acinar and fused gland were the predominant structures. Besides, neoplastic glands invaded the adjacent thyroid follicles to form angular and irregular glands with abundant malignant columnar cells (**Figure 2C**). The cells had copious eosinophilic cytoplasm and round or oval nuclei with obvious chromatin clumping. Extensive angiolymphatic invasion was noticed in the specimen (**Figure 2D**). Mitosis was visible while no necrosis, psammoma bodies, nuclear grooves, inclusions, intracellular bridges, and keratinization were observed. Due to the lack of clear differentiation characteristics of thyroid follicular epithelium and nuclear features of papillary thyroid cancer (PTC), Immunohistochemistry (IHC) analysis was performed. IHC (**Figure 3**) showed diffuse expression of cytokeratin7 (CK7), cytokeratin19 (CK19), thyroid transcription factor-1 (TTF-1), Napsin A, Galectin-3, and BRAF V600E (clone VE1) in tumor cells. About 40% of Ki67-expressing cells were in the hot spot area. However, the specimen was negative for paired-box gene 8 (PAX8), thyroglobulin (Tg), calcitonin (CT), cytokeratin5/6 (CK5/6), and P40. This IHC profile suggested that the lesion was metastatic lung adenocarcinoma (MLA) rather than a primary thyroid carcinoma. Therefore, a clinical-radiological reassessment and biopsy were conducted on the solid lesions in the left lower lung. It turned out that the morphology and immune phenotype of the lung lesions were consistent with those in the thyroid specimen. Furthermore, a *BRAF V600E* mutation was found in both lung and thyroid lesions through Sanger sequencing (**Figure 4**). Accordingly, the lesion was diagnosed as synchronous thyroid metastasis of lung adenocarcinoma with a *BRAF V600E* mutation. The patient ultimately received a 6-month post-operative dual-target therapy with dalafenib and trametinib. The latest follow-up showed the patient was alive 16 months after the initial surgery.

## Discussion

The incidence of thyroid metastasis of cancers is reported to be 0.36% among patients surviving thyroid malignancies (3). However, postmortem studies indicate this incidence reached up to 24% in patients who died with malignancies (4), suggesting that SIT is overlooked or misdiagnosed. Autopsy suggests lung cancers as the predominant source of SIT whereas renal cancer is more frequently identified in clinical labs (3–5), highlighting the challenging SIT diagnosis.

SIT detection is often challenging due to its rarity, asymptomatic nature, and non-specific symptoms. Due to similar sonographic features, high-frequency ultrasound cannot reliably discriminate primary and secondary thyroid lesions, making an accurate diagnosis difficult (11, 12).

If a patient has a clear history of non-thyroid malignancies, SIT diagnosis would be simple. However, SIT might be an initial cancer manifestation in asymptomatic patients without a previous definite diagnosis, as shown in this case. Despite the high diagnostic performance of FNAC for SIT, the absence of specific cytological and histological features makes it difficult to tell between primary thyroid tumors and SIT. Former reports indicate that MLA in the thyroid may resemble the histological and cellular traits of PTC, including papillary structures/papillary-like fronds, nuclear grooves, inclusions, and chromatin clearing (13). Some characteristics, such as prominent nucleoli, coarse chromatins, mitosis, and necrotic background, help distinguish MLA from PTC (13). However, these characteristics cannot distinguish MLA from HGFCTCs (13). Some IHC markers including thyroid follicular cell markers help distinguish primary thyroid tumors from metastatic tumors (3). Markers such as Tg, TTF-1, and PAX8 typically indicate primary thyroid tumors since SITs lack these markers (3). Notably, MLA can demonstrate a significant overlapping immune profile (13). Recent studies have shown that Napsin A is expressed in various thyroid tumor subtypes including thyroid carcinoma (14). Detection of PAX8 and Tg is recommended when it is difficult to distinguish MLA from thyroid carcinoma (13, 14). Multiple studies have reported positive PAX8 staining in several lung cancer specimens using polyclonal PAX8 antibodies rather than monoclonal PAX8 antibodies (13, 15). Some researchers recommend using monoclonal PAX8 antibodies to exclude thyroid metastasis from some cancers, especially lung cancers (3). The useful IHC markers to distinguish MLA from HGFCTC were summarized in **Table 1**. In our case, positive TTF-1 and Napsin A along with negative Tg and PAX8 supported the diagnosis of MLA.

**Table 1:** Summary of the useful IHC markers to distinguish MLA from HGFCTC.

Marker	MLA	HGFCTC
monoclonal PAX8	–	+
Tg	–	+
Napsin A	+	–

IHC, immunohistochemistry; MLA, metastatic lung adenocarcinoma; HGFCTC, high-grade follicular-derived thyroid carcinoma; PAX8, paired-box gene 8; Tg, thyroglobulin; +, posituve; -, negative.

Molecular testing might show signature alterations of primary tumors so it is useful in identifying tumor origins (3). Positive *EGFR* mutations, *KRAS* mutations, and *EML4*::*ALK* fusion in metastatic tumors often suggest a lung origin (3, 13). The *BRAF V600E* mutation is prevalent in thyroid tumors, especially in PTC and HGFCTC but relatively infrequent in NSCLCs. However, in our case, although the *BRAF V600E* mutation was identified in both the lung cancer and thyroid lesion, we can only tell that this mutation drives carcinogenesis rather than identify the primary cancer site because this mutation exists in various malignancies including thyroid cancer and NSCLCs. Since the incidence of the *BRAF V600E* mutation in NSCLC can reach up to 4% (16, 17), combining a meticulous pathologic assessment and ancillary techniques such as IHC would help the diagnosis (3, 13).

Thyroid metastasis management requires personalized strategies that match patients’ health status and tumor stages. Thyroidectomy may be considered for patients with isolated SITs. Radiotherapy or chemotherapy could be efficacious in unresectable cases. IHC and molecular testing not only confirm a SIT diagnosis but also guide treatments if targetable mutations are present. The combinatory therapy using *BRAF* inhibitor dalafenib and *MEK* inhibitor trametinib is promising for patients with *BRAF V600E*-mutated NSCLCs (16, 17) according to the National Comprehensive Cancer Network (NCCN) Clinical Practice Guidelines for NSCLC and the Chinese Medical Association Clinical Diagnosis and Treatment Guidelines for Lung Cancer (18, 19). As demonstrated in this case, dabrafenib plus trametinib substantially and persistently benefits patients with *BRAF V600E*-mutated metastatic NSCLC.

## Conclusion

Accurate SIT diagnosis is critical for clinical management. The lack of a tumor history makes SIT diagnosis challenging. Thyroid metastasis of *BRAF V600E*-mutated lung adenocarcinomas is rare. This case report highlights the importance of a comprehensive diagnostic strategy for SIT diagnosis. Accurate SIT diagnosis relies on a pathologist’s knowledge of cytological and histological features of primary malignancies, as well as the judicious application of a series of combined tests including corresponding immunohistochemical markers and molecular testing. In addition, when encountering atypical tumors that occur in the thyroid, SIT must be included in the differential diagnosis. At this time, clinical doctors should be consulted for medical history or suggestions for further examination can help to prevent potential misdiagnosis and suboptimal treatment.

## Data Availability

The raw data supporting the conclusions of this article will be made available by the authors, without undue reservation.
